# Social media analysis during political turbulence

**DOI:** 10.1371/journal.pone.0186836

**Published:** 2017-10-31

**Authors:** Despoina Antonakaki, Dimitris Spiliotopoulos, Christos V. Samaras, Polyvios Pratikakis, Sotiris Ioannidis, Paraskevi Fragopoulou

**Affiliations:** 1 FORTH-ICS, Heraklion, Crete, Greece; 2 University of Houston, Department of Computer Science, Houston, TX, United States of America; University of Rijeka, CROATIA

## Abstract

Today, a considerable proportion of the public political discourse on nationwide elections proceeds in Online Social Networks. Through analyzing this content, we can discover the major themes that prevailed during the discussion, investigate the temporal variation of positive and negative sentiment and examine the semantic proximity of these themes. According to existing studies, the results of similar tasks are heavily dependent on the quality and completeness of dictionaries for linguistic preprocessing, entity discovery and sentiment analysis. Additionally, noise reduction is achieved with methods for sarcasm detection and correction. Here we report on the application of these methods on the complete corpus of tweets regarding two local electoral events of worldwide impact: the Greek referendum of 2015 and the subsequent legislative elections. To this end, we compiled novel dictionaries for sentiment and entity detection for the Greek language tailored to these events. We subsequently performed volume analysis, sentiment analysis, sarcasm correction and topic modeling. Results showed that there was a strong anti-austerity sentiment accompanied with a critical view on European and Greek political actions.

## Introduction

It is common ground that Online Social Networks (OSNs) have prevailed as the major platform of public expression regarding political matters. Existing studies have performed elaborate analyses in order to investigate the behavior of online users during pre-election periods. The purpose of most of these studies was to generate patterns that distinguish users’ or posts’ favoritism towards one political party or certain ideology. The main predicament in these studies was to generate election predictions that are close or even outperform public opinion polls [1], to measure approval ratings [2] or to assess public opinion during political debates [3]. There exist studies that have tried to measure the emotional content in social media [4]. However, the first term of Barack Obama’s presidency (2009–2012) coincided with the immense increase of Twitter’s user base and its establishment as a channel for personal political expression. As a consequence, one of the first studies that compared sentiment analysis in Twitter with “traditional” opinion polls was from 2010, demonstrating a strong correlation between Sentiment Analysis in Twitter with Obama’s approval ratings polls [2]. The application of the same method in 2012 U.S. presidential elections outperformed the public opinion polls [5]. Since then, numerous other studies have performed similar analysis in other countries like Austria [1], UK [1] and Italy [5] with varying election procedures and diverse cultural and language dynamics.

Perhaps the most seminal review in this area is from Gayo-Avello [6]. This work presents the main considerations in data collection including user and tweet selection, geolocation and language use. It also delineates the main research strategies in the area, which are: (i) classification according to tweet volume, and (ii) classification according to sentiment analysis. Usually, modern studies implement a combination of these two main strategies [7]. Other approaches extract knowledge from the social graph by studying the retweet or mention graph [8] or by averaging on the predefined ideology of the political leaders that the users follow [9]. The tweet volume is a good indicator for a party’s success given that the correct time window is defined [10] but studies indicate that this is inefficient without sentiment analysis [7]. Regarding sentiment analysis techniques, researchers use specially tailored dictionaries with positive, negative or neutral colored words, and measure the occurrence of these words in a rich variety of language properties of the posted text [11, 12] or hashtags [13]. Today, sentiment analysis is routinely used even for real-time analysis [14]. Gayo-Avello [6] also lists the major difficulties of this area that need to be addressed before making Twitter a reliable election prediction mechanism. In brief, these difficulties are noise and demographics.

Regarding noise, a huge proportion of election-related Twitter posts are humorous, ironic or sarcastic and do not portray any party (or ideology) inclination. It is estimated that approximately half of collected tweets belong to this category [15, 16]. Filtering out these posts or users is a challenging task and relies heavily on qualitative human-crafted datasets of sentiment vocabularies and pre-classified “ground truth” samples [17]. Low-quality human-curated dataset can result in a very inefficient classification algorithm, as it happened in a sarcasm detection system [18]. Existing studies on sarcasm detection are focusing on user and word selection techniques [1], or are explicitly addressing reliability level of posts by classifying them as rumors or trolls [19].

Regarding demographics, Twitter users belong to a specific social group that is not necessarily representative of the whole electorate. Specifically, studies have indicated that Twitter users belong to a certain age [20], social [21] and ideology demographic group and, therefore, express a partial opinion of the society at best. A study of 2011 concluded that, due to its demographics, Twitter is by far inferior compared to opinion polls for elections prediction in the U.S. [20]. Another study reported that existing political party classification systems, based on sentiment analysis, are no better than random classifiers [22]. This indicates that sentiment analysis methods are in their infancy and that they should be coupled with more sophisticated methods that incorporate rich lexical properties and context indicators specific to each campaign [7]. Fortunately, existing techniques can effectively assess and correct these biases [21].

This work aims to apply natural language analysis techniques on Twitter data related to two electoral events that happened on 2015, during a politically turbulent period of Greece that was triggered by an effort to negotiate a reconstruction of its national debt. These events were the Greek bailout referendum that took place at 5 July 2015 and the second was the subsequent legislative elections that took place at 20 September of the same year. The purpose of this study is to identify the tweeting patterns, the expressed sentiment and the semantic relations of the most important entities that prevailed during the online discourse that preceded these two events.

To accomplish this we have split our analysis in 5 distinct parts. The first is data collection and Entity Identification (EI). EI is the process of extracting the most important notions (entities) that are prevalent in users’ posts. Examples of extracted entities are ‘Prime Minister’ and ‘Debt’. After EI, each entity is represented by a set of words with equal meaning (i.e ‘EU’ and ‘European Union’). Although there is a variety of methods for automatic EI, they are all inferior to various extend to human curators [23]. For this reason and given the relative narrow semantic context (elections) of our dataset this task was performed manually. Having in our disposal a set of prevalent entities we proceeded to perform Volume Analysis. Volume Analysis of tweets simply studies the count differences between tweets that belong to certain entities. There are contradicting studies regarding the predictive ability of tweets count for election results (for examples of positive findings see [24, 25] and for a negative see [26]). Nevertheless most studies agree that tweet count can give valuable information if not for the election outcome then for the quantitative estimation of the political inclinations of Twitter’s user-base. For this reason we apply Volume Analysis in the referendum dataset because it has a simpler structure (only two choices: YES/NO). We found that indeed tweet counts matched the referendum results and we also associate changes in the temporal variation of the ratio between ‘YES’ and ‘NO’ tweet counts with real events. The third part is the study of Entity co-occurrence where we visualize in 2-D space entities by simulating a graph of spring forces. On this graph, the higher the number of co-occurrence between two entities the stronger the force. This method has been used in the past to visualize (among other) semantic data [27] and online social networks [28] but it has not been used, to our knowledge, to visualize Twitter extracted entities. This is a very computationally efficient method that gave insights on the ‘NO’ and ‘YES’ affiliated entities when applied to the referendum dataset. On the fourth part we apply sentiment analysis which is perhaps the most notable collection of methods for analysis of textual content that is rich of human opinions [12, 29]. The novelty of our approach is that we use a novel sentiment dictionary for the Greek language and that we also account for the presence of sarcasm that has been found to severely confound sentiment analysis [30]. The temporal variation of sentiment for various entities along with the identification of the most and least sarcasm-prevalent entities provided additional insights on user’s opinions. Finally the fifth step is topic modeling which is an unsupervised learning method that estimates the probability of an entity to belong to a distinct cluster, also called ‘topic’. Each topic is an automatically-extracted semantic structure of the input corpus. Through topic modeling we can extract the hidden semantic similarities of our data and visualize their proximities in 2-D space. Applying topic modeling algorithms like Latent Dirichlet Allocation in Twitter data entails certain difficulties due to the brevity of text messages [31] that we were able to overcome by applying sarcasm correction. Topic modeling revealed that ‘YES’ and ‘NO’ entities were unexpectedly close in the referendum dataset. It also spatially outlined the relationships of political parties that took part in elections.

Applying these methods to Greek tweets entails some additional difficulties. First, the online language, primarily used by the youth, is a mix of Greek grammar with Latin letters. This system, called “greeklish”, stems from early text-based communication systems that had limited support for Greek letters. Moreover, this system has not any standard correspondence between Greek and Latin letters, whereas it often disregards Greek grammar and punctuation. The result is a highly complex language with multiple possible writings even for basic and short words that makes automatic detection a very tedious task [32, 33]. Although nowadays the majority of Greek users are using Greek characters when tweeting, most of the included hashtags are present in a “greeklish” form. Additionally, the demographic subset of Greek Twitter users is narrower than in other western countries thus limiting its representative power [16]. Nevertheless, Charalampakis et al. [16] were able to perform irony detection in Greek political tweets and inferred similar percentages with studies focusing in U.S. politics.

### Political background in Greece

On 25th of January 2015, the new “anti-austerity” government of the SYRIZA party was elected in Greece with a percentage of 36.3%, starting a long negotiation with the Eurogroup about debt reconstruction. Until June 2015, there was no visible progress achieved and SYRIZA decided to throw a referendum on 5th of July 2015 so that the Greek people decide whether to accept or not the current austerity measures proposed by the Eurogroup. Capital controls were enforced in Greece and the result of the referendum was NO (do not accept) with a percentage of 61.3%. Eurogroup did not accept the result of the referendum as a bargaining tool and, under extreme pressure, the government decided to accept the proposed measures. Several disagreeing members of the SYRIZA party threatened to vote down the measures. The prime minister (Alexis Tsipras) decided to expel the disagreeing Members of the Parliament that belonged to the governing party, and announced new legislative elections on 20th of September 2016. SYRIZA won the elections again with a reduced percentage of 35.5% and the party formed mainly from disagreeing members (called LAE) did not get enough votes to enter the parliament.

## Materials and methods

### Twitter corpus

**Fig 1:**
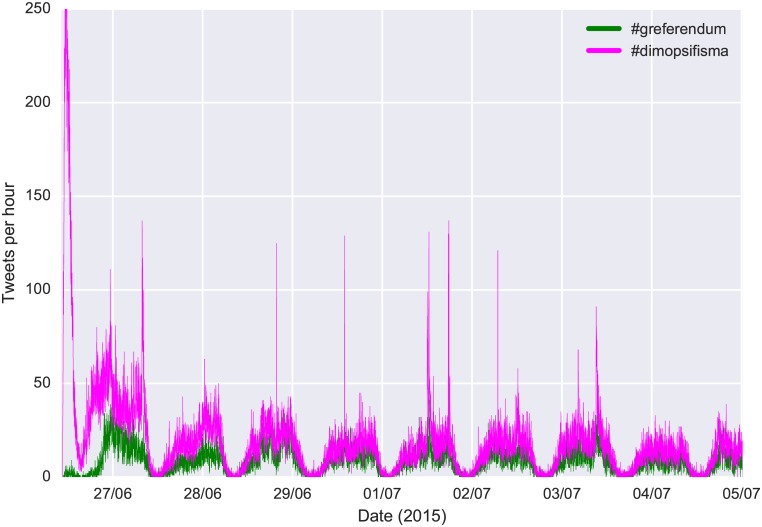
Frequency of referendum tweets per hour. The frequency of tweets peaked right after the Referendum was announced and followed a small declining trend. The day/night patterns are also visible.

Our analysis is based on two distinct Twitter datasets. The first one includes all tweets that contain the #dimopsifisma and #greferendum hashtags. These were by far the most prominent hashtags that prevailed throughout the period that preceded the Greek bailout referendum (“dimopsifisma” is the Greek word for referendum). This period was from 25th June 2015 when the referendum was announced, until 5th July 2015 when the referendum took place. Data were collected through Twitter’s API. This dataset contains in total 301,000 tweets out of which 84,481 are neither retweets nor replies. The frequency of referendum tweets is illustrated in Fig 1, where we notice the day and night patterns as well as a decline of tweets frequency over time. The second dataset includes all tweets that contain the hashtags #ekloges and #ekloges_round2 (“ekloges” is the Greek word for elections). These hashtags dominated the online discussion regarding the Greek legislative elections that were announced on 20th August 2015 and were held on 20th September of the same year. In total, this dataset contains 182,000 tweets out of which 45,750 are neither retweets nor replies. In essence, these two datasets contain the complete online discourse that happened in the Greek Twittersphere regarding the two political events (referendum and elections). Only tweets with at least one Greek letter were included in our analysis. Since these events had attracted a worldwide interest (especially the referendum), this filtering is essential to eliminate content not representing the Greek electorate. The complete analysis took place in a cloud infrastructure.

### Entity identification

To support our analysis and reveal relationships between persons, institutions, events and abstract notions (such as democracy or liberalism), we performed entity identification [34] on the elections and referendum Twitter corpus. As a first step, we gathered all unique words and Twitter hashtags present in the tweets along with the respective occurrence frequency of each. Then, we manually selected all entities relevant to the political domain of the Greek legislative elections and referendum of 2015, apparently considering the most frequent words and hashtags as of higher importance. Afterwards, we grouped all various forms that a given entity appears in, so that we would be able to identify a certain entity regardless of the variant it appears with in the tweets. For example, all of the following hashtags identify a single entity, that of the Greek Prime Minister, Alexis Tsipras: #Tsipras #atsipras #alexistsipras #atsipra #aleksitsipra. We performed grouping of variants for entities found either as plain text in the tweets or mentioned as hashtags. In total, we extracted 156 entities from plain text of tweets and 116 entities from Twitter hashtags; the minimum, maximum and average number of variants per entity is listed in Table 1. Before matching entities appearing in hashtags and in the tweet text, we performed a normalization of all tweets in order to minimize variation coming from common spelling mistakes. For this purpose, we grouped commonly misspelled diphthongs and punctuation into a single form. Subsequently, we linked and combined occurrences of a given entity that appeared both as plain text and as hashtag to improve precision of entity identification. Lastly, for each tweet, we located all entities that are referenced either as hashtag or as plain text, distinctively, and annotated our dataset accordingly for further processing. This laborious manual task was necessary in order to import semantic knowledge around the context of these events into our analysis.

**Table 1:** Entity variants from plain text and hashtags.

	Text Entities	Hashtag Entities
Number of Unique Entities	156	116
Min Number of Variants per Entity	1	1
Max Number of Variants per Entity	74	148
Average Number of Variants per Entity	18.9	21.6

### Sentiment analysis

For sentiment analysis, we used SentiStrength [35] that is ideally suited for the affective dimension of the social web and Twitter in particular [36]. Texts often contain a mix of positive and negative sentiment and for some applications it is necessary to detect both simultaneously and also to detect the strength of sentiment expressed. SentiStrength employs several methods to simultaneously extract positive and negative sentiment strength from short informal electronic text. The main power of SentiStrength is in the combined effect of its rules to adapt to various informal text variations as well as in the overall approach of using a list of term strengths and identifying the strongest positive and negative terms in any comment. SentiStrength introduces a dual 5-point system for positive and negative sentiment. More specifically, it reports two sentiment strengths associated with a given piece of text: -1 (not negative) to -5 (extremely negative), and 1 (not positive) to 5 (extremely positive). SentiStrength uses two scores because humans process positive and negative sentiment in parallel. Positive and negative sentiment can coexist within texts and so it also seems reasonable to conceive sentiment as separately measurable positive and negative components. For example, the text “I love you but hate the current political climate.” has positive strength 3 and negative strength -4.

### New lexicon for sentiment strength detection

Sentiment analysis is known to be domain-dependent, meaning that applying a classifier to a dataset different from the one on which it was trained often gives poor results [37]. Indeed, the diversity of topics and communication styles in the social web suggests that many different classifiers may be needed. Existing general-purpose social web sentiment analysis algorithms may not be optimal for texts focused around specific topics, such as the political domain in our case. Indeed, a major weakness of SentiStrength is that its general sentiment lexicon performs poorly and achieves very low accuracy in political texts. However, SentiStrength supports topic-specific lexicon extension, which involves adding topic-specific words to the default general sentiment lexicon [38].

Therefore, we enriched SentiStrength for the Greek political domain by creating new general-purpose and political-domain lexicons through manually selecting and annotating words from the Twitter corpora. Human intervention seems likely to be particularly important for narrowly-focused topics for which small misclassifications may result in significant discrepancies if they are for terms that are frequently used with regard to a key aspect of the topic. For the purpose of political domain analysis, we manually created a new SentiStrength-compatible lexicon comprising Greek words with associated positive/negative sentiment strength, aiming to improve the accuracy and effectiveness of political-domain lexical sentiment strength detection. The SentiStrength algorithm for sentiment strength detection across the social web primarily uses direct indications of sentiment. Since our study is domain-dependent and time-dependent (political domain and Greek legislative elections and referendum of 2015, respectively), we included indirect affective words too, in order to enhance sentiment detection. These words identify terms that associate with sentiment but do not directly express it, and were derived from the Twitter corpus in question.

The new sentiment detection lexicon we compiled, is a merge of the following 3 lexicons in Greek: (i) SentiStrenth’s built-in lexicon that provides general sentiment analysis for the Social Web; (ii) SocialSensor lexicon that is utilized by SocialSensor framework to collect, process, and aggregate big streams of social media data and multimedia to discover trends, events, influencers, and interesting media content in real time [39]; and (iii) our new political-domain lexicon that introduces lexical sentiment strength detection for political texts, and is based on frequently used terms in the elections and referendum Twitter corpus. For a given topic there may be rare words or specialist words that are frequently used to express sentiment. The lexical extension method we applied, identifies these words and uses them to improve sentiment strength prediction through a political-domain lexicon extension (i.e., a set of words and word strengths). The size of lexicons expressed as number of words contained, can be seen in Table 2. Last but not least, SentiStrength allows for insertion of wildcards (an asterisk character *) at word stems in the lexicon, thus covering the word inflections. Therefore, we have made extensive use of wildcards in the newly created sentiment strength detection lexicon. This feature is extremely useful to enhance word matching and is particularly suited to Greek language morphology, since Greek is a highly inflected language.

**Table 2:** Number of words contained in lexicons.

	Number of Words
SentiStrength	1638
SocialSensor	2315
Domain-Specific	974
New Lexicon	4915

### Sarcasm detection

As we have discussed, a significant percentage (∼50%) of tweets referring to political issues are of sarcastic or humorous nature and can severely obscure the analysis, particularly in the political domain [40, 41]. To detect this content, we adapted the method used by the online sarcasm detection service, [42], in order to be able to characterize Greek text.

The first step was to construct a sarcasm classification mechanism [18, 43]. Initially, we built a database that contained all original tweets. These were the tweets that were neither retweets nor replies. Out of the total 483,000 tweets belonging to the referendum and election datasets, we extracted 130,231 original tweets. Subsequently, we built a website that showed random tweets and users got to choose whether each tweet was sarcastic or non-sarcastic/normal. Users could also skip a tweet in the case that they could not make a safe decision. In order to assure uniform classification from many human judges, the website also explained the context of the study including a simple explanation of “sarcasm” in Twitter. We promoted the website through social media and after a week we collected the human-flagged tweets. In total, it contained 2,642 sarcastic tweets and 2,002 negative non-sarcastic/normal tweets from 134 different user sessions.

Having at our disposal a human-flagged dataset regarding sarcasm, we proceeded to build a classification model. From the total 4,644 flagged tweets, we extracted lexical and semantic features. In order to build the lexical features, we developed a stemmer for the Greek language and we built a stopword collection containing commonly used Greek words. Then, we extracted 1-grams and 2-grams for each tweet. A common set of lexical features is Part-Of-Speech (POS), which we were not able to incorporate since we could not locate an adequate POS dictionary for the Greek language. The semantic features included average sentiments for each word in the tweet and topics. For sentiment, we used the same SentiStrength-compatible dictionary that we constructed for the purposes of this study. We also performed a topic analysis that generated 100 topics related to the context of the collected tweets. The hypothesis here is that some topics are more associated with sarcastic tweets and therefore, by incorporating them as features, we can improve the classification efficiency of our model. The topic analysis method that we used was Latent Dirichlet Allocation (LDA) implemented with the Gensim Python library. For classification, we used a Support Vector Machine (SVM) classifier with a linear kernel and an Euclidean regularization coefficient of 0.1. Subsequently, we randomly divided the flagged dataset into 70% training dataset and 30% test dataset. We trained our model to the training dataset and estimated its performance on the test dataset. The classification results are on Table 3.

**Table 3:** Classification results.

	Precision	Recall	f1-score	Test Samples
Non-Sarcastic	0.69	0.62	0.65	621
Sarcastic	0.72	0.78	0.75	772
Average/total	0.70	0.71	0.70	1393

For comparison, Charalampakis [16] performed exactly the same task (sarcasm detection in Greek tweets regarding politics) and reported 80% True Positive Ratio (TPR), but with extremely low number of samples (126). Our TPR estimate of 0.78 for sarcastic tweets is similar to estimates from other studies, such as 0.71 by González-Ibáñez [18] and 0.75 by Liebrecht [43]. Since “sarcasm” is a subtle and ambiguous notion, especially in the political context, it is questionable whether significantly superior results are possible. This conclusion is supported by the fact that even humans have a limited ability to detect sarcasm in Twitter that ranges from 70% [18] to 85% [43].

Using the trained SVM classifier, we generated “sarcasm values” for all 130,000 original texts in our dataset. These values are in the form of percentages that range from -100% (definitely not sarcastic) to 100% (definitely sarcastic). For each tweet the SVM classifier calculates a confidence score which is the signed distance of that tweet from the optimal hyperplane calculated during training. We then applied the hyperbolic tangent (tanh(*x*)) as a sigmoid function to convert this distance to percentages. Finally mapped each “sarcasm value” to one of the following categories: “no_sarcasm” for negative values, “sarcasm_1” for values from 0% to 20% of positive sarcasm values, “sarcasm_2” for values from 20% to 40% of positive sarcasm values, and “sarcasm_3” for values greater than 40% of the “sarcasm value”. In our public repository [44] we show some examples of tweets belonging on these categories.

Sarcasm detection revealed interesting indirect affective words. These are words that were used mainly in sarcastic tweets for mocking or ironic purposes. The top indirect affective words were ATM (due to Capital Controls, people could withdraw a limited amount of cash through ATMs), Hope (used in SYRIZA’s slogans), Merkel (Germany’s Chancellor), memorandums (sets of austerity measures imposed by EU), bankruptcy, drachma (Greece’s currency before Euro) and recovery.

## Results

### Tweets’ volume analysis

**Fig 2:**
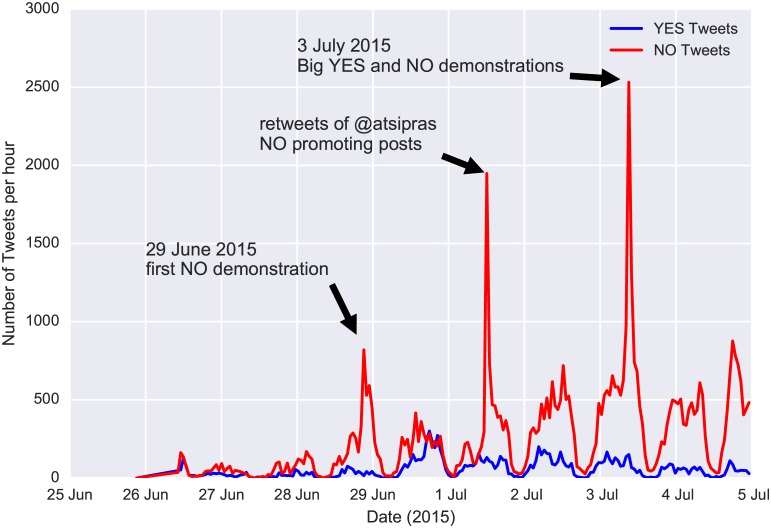
Frequency of YES/NO tweets in referendum. The number of “NO” tweets were persistently higher than “YES” tweets throughout the pre-referendum period. Also certain “NO” promoting tweets and events generated a public sensation that are visible as spikes in the red line.

**Fig 3:**
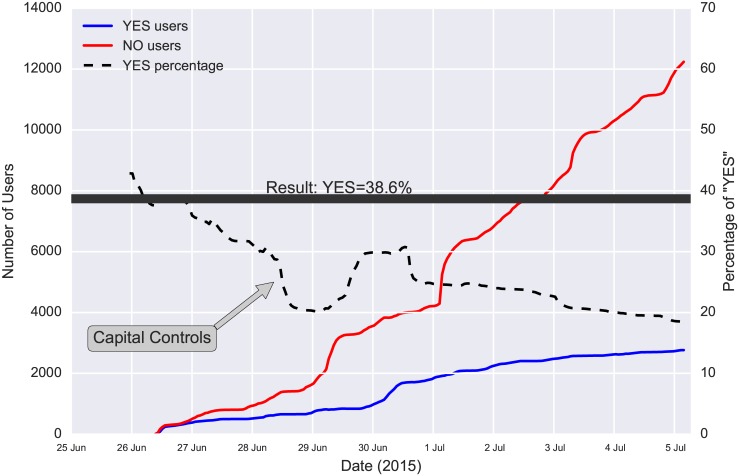
Variation of YES percentage. Red and blue lines represent the cumulative number of users that have posted exclusively “YES” and “NO” tweets respectively for each time point of the pre-Referendum period. The black dashed line is the “YES” to “NO” user ratio and the solid black line is the final “YES” percentage (38.6%). Perhaps unexpectadly the enforcement of capital controls strengthened temporarilry the “NO” percentage.

**Fig 4:**
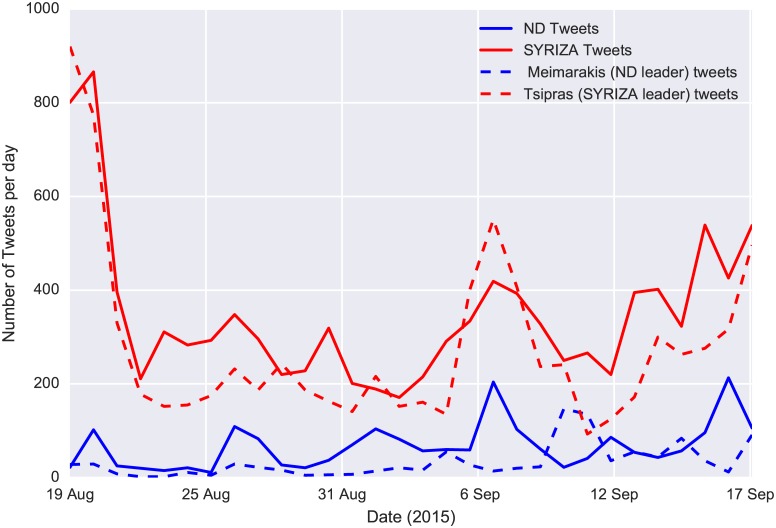
Frequency of election tweets. Immediately after the announcement of Elections the volume of tweets referring to the leading party (SYRIZA) and its leader (Alexis Tsipras) started a decreasing trend. Nevertheless this volume remained higher than the tweets referring to the main opposition (ND) and its leader (Meimarakis).

Although the tweets’ volume is not a sufficient indicator of political inclinations of users, it can give insights regarding specific events. In Fig 2 we plot the volume of referendum tweets per hour. We focus only on tweets that contain either *voteYES* or *voteNO* entities. The spikes in this plot are indicative of major events during the pre-referendum period. Analysis of the text from these tweets revealed that they were either prompting people to participate in certain demonstrations or they were retweets of the prime minister, urging for “NO” votes. Fig 2 also shows the decreasing temporal variation of the ratio of users who included “YES” vs. “NO” entities in their tweets. Interestingly, opinion polls that were conducted during the same period showed an opposite trend, which, according to post-referendum analysis, was erratic [45]. The final “YES” vs. “NO” ratio right before the referendum was 18%, which, despite the high difference from the final result (38.6%), was very close to the preferences of the demographics of Greek Twitter users. According to [46], users belonging to the age groups of 18–24 and 25–34 voted “YES” with a percentage of 15% and 27.7%, respectively. In Fig 3 we also observe the effect of Capital Controls on the “YES” vs. “NO” ratio. Perhaps unexpectedly, the enforcement of Capital Controls temporarily strengthened the “NO” sentiment. The volume of tweets that were referring to the leading party (SYRIZA) and its leader (Alexis Tsipras) had a decreasing trend during the pre-elections period (Fig 4). In contrast, there was a slight increase in the volume referring to the SYRIZA’s major opposition party, New Democracy (ND). Additionally, the total number of the pre-elections tweets (180,000) that lasted for one month, was significantly lower than the pre-referendum tweets (308,000) that lasted for only one week. This is supported by the fact that the elections turn-out was exceptionally low (56.6%). So, although there were not very strong variations in sentiment, the tweet volume forms a good indicator of the general enthusiasm or apathy feeling towards the elections. Additionally, when the predicament of a referendum is simple (like a “YES”/“NO” question), the tweet volume can give a precise estimation of the final result for the demographics that Twitter represents.

Another interesting question regarding tweets’ volume is the potential existence of different tweeting pattern between “YES” and “NO” voters. To investigate this we measured the average tweets posted by “YES” users and “NO” users. We define as “YES”, a user that has posted at least one “YES” entity and none “NO” entity. Similarly, we define “NO” users accordingly. In total, our dataset had 1.558 “YES” users and 11.672 “NO” users. In our public repository [44] we show some examples of “NO” and “YES” tweets. Yet, in average, “YES” users posted approximately twice as many tweets (11.3) than “NO” users (6.1). We applied a Mann–Whitney U test which is a non-parametric test with the null hypothesis that a random sample from the “YES” users set will have equal probability of having less or greater number of tweets from a random sample in the “NO” users set. The p-value (5.3 * 10^−96^) indicates a strong correlation between voting preference and number of tweets. If we limit our analysis in tweets that contain only “YES” or “NO” entities then, in average, “YES” users posted 2.1 tweets with the “YES” entity and “NO” users posted 1.7 tweets with the “NO” entity (Mann–Whitney U test p-value = 4.1 * 10^−6^). This finding is suggestive that “YES” users were engaged in an orchestrated campaign to promote “YES” content. The deliberate use of bots or real conscripted users to promote a particular ideology or party prior to an election event (also called “slacktivism”) is a known phenomenon [47]. Validating this phenomenon and measuring its effect is a challenging task that we include in our future work.

### Entities co-occurrence

**Fig 5:**
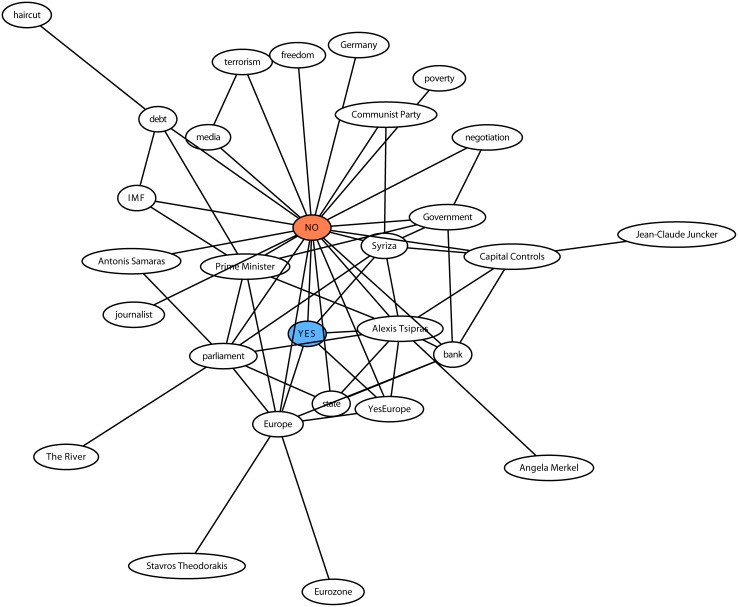
Entities co-occurrence in referendum. This graph shows the force-directed graph drawing of main entities (more than 500 tweets) of the Referendum dataset. The distance between two entities represents the number of tweets in which they co-occur (the higher co-occurrence the closer the distance). On the upper part (closer to “NO”) are mainly anti-austerity entities and on the lower part (closer the “YES”) are pro-european entities.

Two entities are co-occurring if there exists at least one tweet that contains both entities. We define distance between entities as: *d* = *log*(10 + *c*_*max*_ − *c*), where c is the number of tweets that contain a specific pair of entities, and *c*_*max*_ is the maximum c (max co-occurrence). We apply the “neato” visualization method of Graphviz software, which emulates spring link attractive forces between nodes [48]. In Fig 5 we visualize the distances of entity pairs with at least 500 occurrences for the referendum dataset. In this figure we notice that *YES* and *NO* entities are central to the discussions with a small in-between distance. Moreover, it is clear that Europe-related entities are closer to the *YES* point, whereas entities regarding domestic affairs including *debt* are closer to the *NO* point.

### Sarcasm, sentiment and hashtags

Sarcasm detection revealed some points of interest pertaining to the use of sarcasm in the political domain. Overall, 61.8% of the total referendum tweets and 58.7% of the total election tweets had a positive sarcasm value (>0%). Nevertheless the percentage of tweets with strong sarcasm (>20% sarcasm value) was 27.1% and 28.8% for referendum and elections tweets respectively. A plot of the Cumulative Distribution Function of the sarcasm values is included in our code repository [44]. For comparison a study that involved sarcasm detection in 2012 US elections, found 23% sarcastic tweets [49]. This percentage is similar to the 29% that was detected in a collection of tweets regarding the candidates of the Republican party who were running for the US Presidential nomination for the same elections [50]. Nevertheless, the only other study (to our knowledge) that attempted to identify sarcasm in greek political tweets, was performed in a much smaller (44,000 tweets) dataset referring to the Greek legislative elections of 2012 and concluded that 54.5% of tweets are sarcastic [51]. Since the subject of a tweet is a very strong indicator of sarcasm it is difficult to obtain a ground-truth regarding sarcasm percentages. For example, according to [52], sarcasm percentages of tweets vary from 3% to 85% according to their associated topics and ‘Politics’ is one of the most sarcasm-prevalent ones. A comparison study of existing sarcasm detection methods tailored for Greek political tweets can shed more light on this and it is included in our future work.

**Fig 6:**
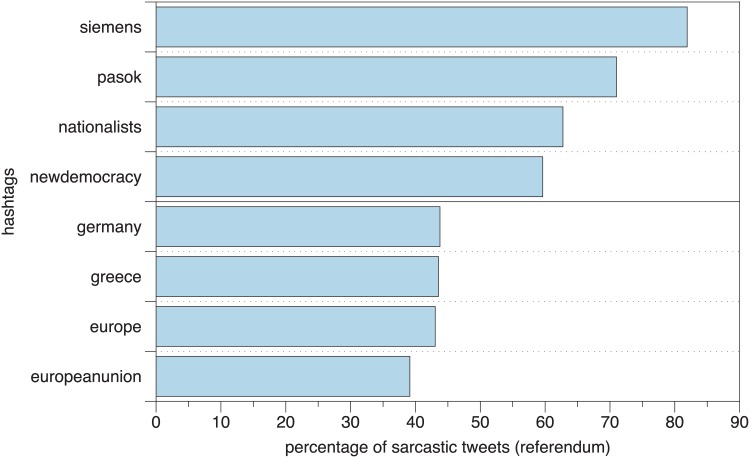
Hashtags mainly used in sarcastic and non-sarcastic posts during the pre-referendum period. A sarcastic tweet is defined as one having positive sarcasm values whereas non-sarcastic tweets have negative sarcasm values.

Ironic posts were prevalent for specific hashtags, which, after looking into the text entities, revealed the level of the citizen aversion to the entities involved in the current situation, namely, the earlier governments and a company in the center of talk about corruption (Fig 6). On the other hand, the least use of irony was found to feature the talk about the entities at stake that would be affected the most by the referendum outcome, such as Germany, Greece, Europe, and the EU.

**Fig 7:**
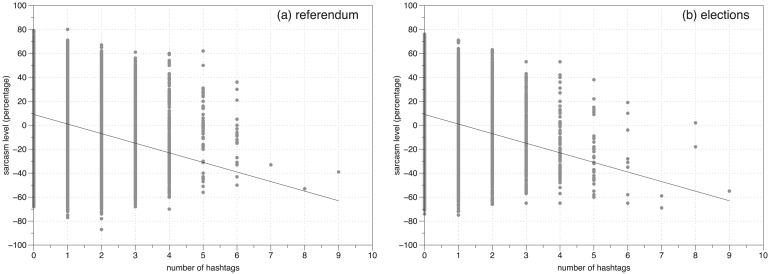
Number of hashtags and sarcasm. Each point in these figures is a tweet. Figs (a) and (b) contain tweets in Referendum and Elections respectively. X axis contains the number of hashtags. The ‘sarcasm value’ (y axis) ranges from -100% (definitely not sarcastic) to +100% (definitely sarcastic). Tweets with high number of hashtags exhibit lower values of sarcasm.

In both referendum and elections data, it is also worth noting that a negative correlation exists between sarcasm and number of hashtags Fig 7. The Fisher Transformation Test for referendum was, *z* = 43.225, *p*<0.001 and for elections, *z* = 34.839, *p*<0.001.

**Fig 8:**
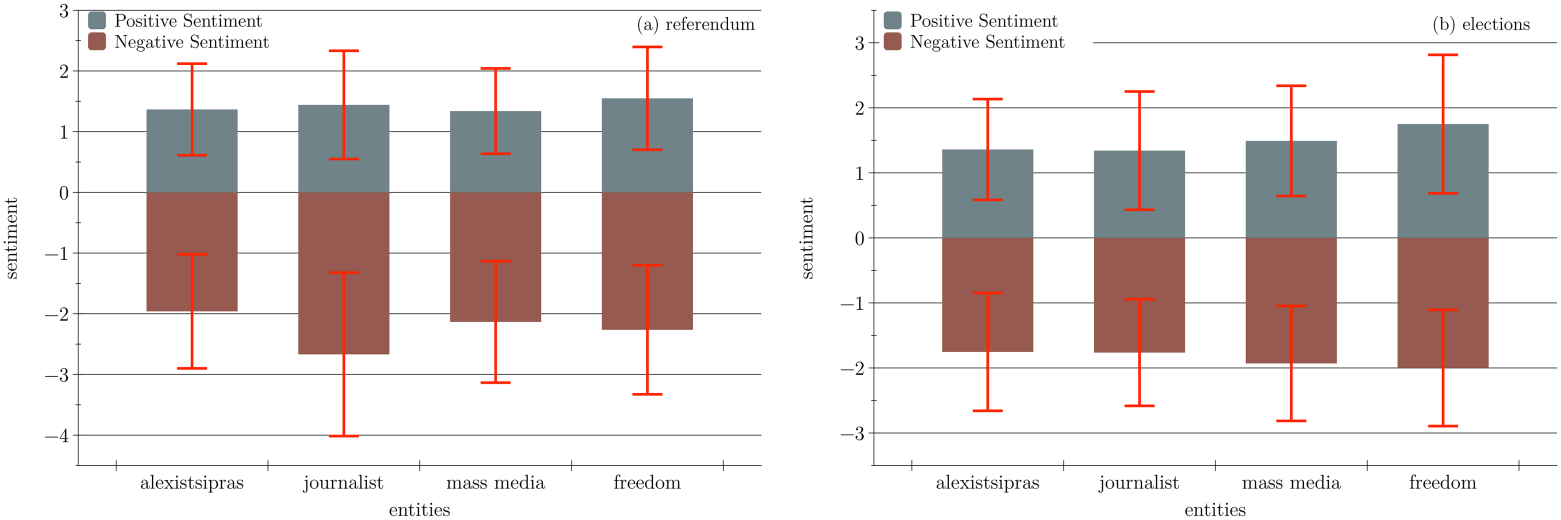
Entities with extreme sentiment polarity. Figs (a) and (b) show the entities that exhibit the highest sentiment polarization during the pre-Referendum and pre-Elections period respectively. Polarization is measured as the difference between the average positive and negative sentiment. The negative sentiment values have higher range in Referendum (from -4 to 0) than in Elections (range from -3 to 0). Sentiment values (y axis) are measured according to the SentiStrength score.

Although the sarcasm assignment provided a glimpse into the thoughts of the citizens revealing causes and worries related to the outcome of the referendum, there was a different but equally valuable aspect exposed by the sentiment polarity. Looking at the entities that exhibit the highest polarization of sentiment (defined as the difference between the average positive and negative sentiment values for each entity), one can notice how the citizens thought about the forces that actively tried to influence the outcome of the referendum (Fig 8). By retrieving the tweets mentioning more than one of the highlighted entities, it was found that extreme polarization could be seen in those texts, clearly separating the negative sentiment towards *journalists* and the *mass media* against the positive sentiment towards *Alexis Tsipras* and *freedom*. For the elections, although new extreme positives and extreme negatives emerged (such as *terrorism* and *poverty*), the same four entities exhibited the highest polarization. Revealing the entities that exhibited the highest polarization of sentiment provided and examining the words that carried that sentiment, one may observe the actual cause of polarization. Upon examining the content of the referendum, the citizens perceived the journalist and mass media input as propaganda, an attempt to steer the citizens to vote for specific pro-austerity parties. Both analyses provided clear indication of the citizen perception of journalists and the mass media contribution to both electoral events. This was further reinforced upon examining the co-occurrence with the remaining two highly polarized entities. The negative sentiment co-occurred in the context of the propaganda while the positive in the context of establishing freedom through voting for the prospective candidate. Co-occurred sentiment polarization provided insight to the connections the citizens perceived and justified towards their elections voting.

Another feature across both datasets is the correlation between sentiment and sarcasm. With regard to the referendum data: a positive correlation exists between positive sentiment and sarcasm, Fisher Transformation Test, *z* = 17.137, *p*<0.001; and a negative correlation also exists between negative sentiment and sarcasm, Fisher Transformation Test, *z* = 15.954, *p*<0.001. Similarly, for the elections: a positive correlation exists between positive sentiment and sarcasm, Fisher Transformation Test, *z* = 13.508, *p*<0.001; and a negative correlation exists between negative sentiment and sarcasm, Fisher Transformation Test, *z* = 19.719, *p*<0.001.

### Temporal variation of sentiment

**Fig 9:**
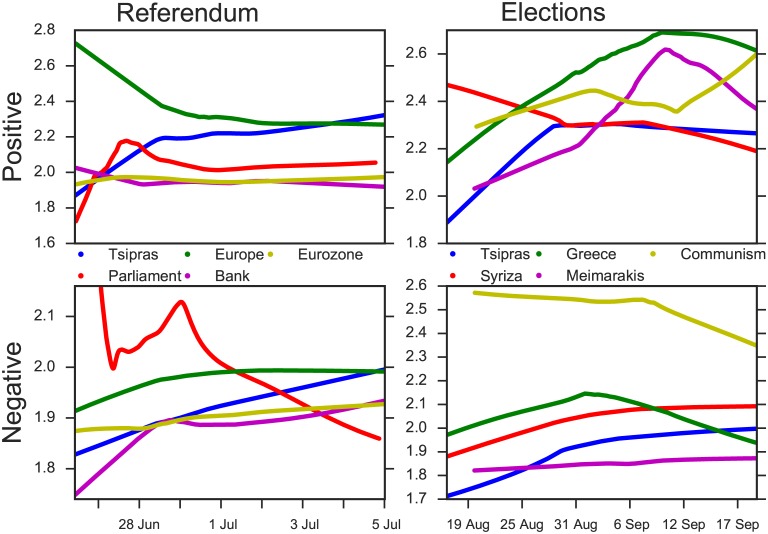
Variation of sentiment in referendum and elections. Local Linear Regression Lines (LOESS) for the top 5 most frequent entities of Referendum and Elections. Sentiment (y axis) is encoded according to SentiStrength. Positive sentiment ranges from 1 (not positive) to 5 (extremely positive). Negative sentiment ranges from -1 (not negative) to -5 (extremely negative).

The computation of sarcasm and sentiment levels allows us to plot the temporal sentiment variation for any entity. To eliminate the influence of sarcasm, we applied “sarcasm correction” to the sentiment for tweets with positive sarcasm. Specifically, each tweet sentiment was corrected towards the neutral side proportionally to the percentage of sarcasm that it contained. In Fig 9 we show the local linear regression lines (LOESS) of positive and negative sentiment over time for the top 5 most frequent entities of referendum and elections. The sentiment (y axis) is encoded according to SentiStrength [35]. From this plot we notice that during the pre-referendum period, the positive sentiment for Europe decreases and the negative sentiment for the Greek Prime Minister Alexis Tsipras increases and becomes almost stable after the enforcement of the Capital Controls on June 29th. Regarding the elections, this trend is reversed since the leading party, SYRIZA, undergoes a decrease in positive sentiment and an increase in negative sentiment. This demonstrates a general dissatisfaction of the party actions regarding the post-referendum political developments.

**Fig 10:**
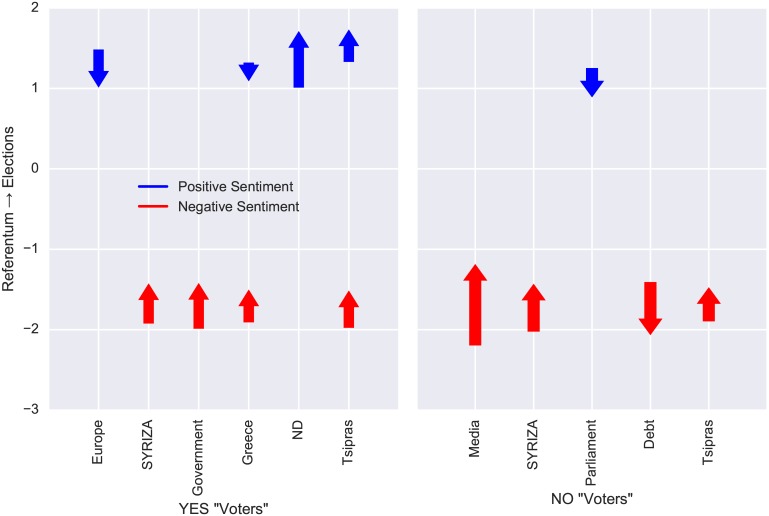
Change of sentiment between referendum and elections. The arrows show statistically significant changes in the average sentiment between referendum and elections for the same group of users (“YES” voters and “NO” voters). Negative values represent negative sentiment, while positive values represent positive sentiment. A greater absolute value for a negative or positive sentiment, signifies that the sentiment is more intense.

After the referendum, the government did the highly criticized action to accept Eurogroup’s measures despite the high percentage of the “NO” vote. We expect that this move generated many sentiment shifts for various entities. Therefore, it is interesting to examine how “YES” voters and “NO” voters reacted to this development. To measure this, we split users into two disjoint groups: the “YES” voters and the “NO” voters. We also kept only users that have posted in both referendum and elections datasets. For each user in every group, we measured the average positive and negative sentiment for each entity in both datasets. Finally, for each entity, user group and sentiment, we applied the Mann-Whitney U test between the average sentiment of this group in the referendum dataset and the average sentiment of this group in the elections dataset. In Fig 10 we show entities for which the sentiment was significantly changed (*p* < 0.001). The direction and length of the arrows in the figure portray the vectors of this change. In this figure, we notice a general shift of negative sentiments towards neutrality (the only exception is the “Debt” entity for the “NO” voters). Indeed, elections (which constitute a more frequent electoral event), did not attract the same negatively charged content. Moreover, after the referendum, “YES” voters expressed more positive comments regarding “ND” (i.e., the main opposition party) and the prime minister Alexis Tsipras.

### Topic modeling

Topic modeling is a powerful method to detect thematic patterns in text corpus. Applications in political discussions can reveal interesting ideological inclinations, tendencies and concept proximities. One of the most frequently used techniques in this area is Latent Dirichlet Allocation (LDA), which has been used in the past to analyze online content, like news items and blog posts, and for spam detection.

Topic modeling generates a predetermined number of topics. For each topic it computes a per-entity distribution, or else the probability that an entity belongs to a topic. Additionally, topics can be projected in a 2-dimensional space for better visualization. As a result, two topics lying in distant places after LDA analysis, indicate that they have very different mixture of entity probabilities. In contrast two proximal topics indicate a concordance of entity probabilities.

This method has also been used in the past in order to analyze political content in Twitter. During the German federal elections of 2013, LDA was used to quickly identify emerging topics [53] in Twitter. This method was able to detect prevalent discussion topics earlier than Google Trends. Another study analyzed the extracted topics from tweets regarding Barack Obama [54]. Consequently, the authors, applied content summarizations methods in order to locate the most insightful opinions in each topic. In our case we want to study the semantic distance between prevalent opponent entities in both Referendum and Elections. While sentiment analysis reveals the overall positive and negative emotions that characterize each entity, topic modeling reveals the entities that exhibit semantic similarity.

The short length of Twitter posts, the special linguistic elements that they contain, and the variability of the political discussion, make the application of LDA in political-related Twitter content difficult [31]. Here, we argue that performing entity identification combined with sarcasm filtering, we can efficiently locate dominant topics in Twitter.

For our study, we analyzed the manually-identified entities in the tweets with Gensim Python library, after excluding all tweets that had sarcasm identifier value higher than 5%. For visualization of the generated topics, we used LDAvis [55] that performs a Principal Component Analysis (PCA) to project the identified topics on the 2-dimensional space. Moreover, for each entity, we measured the average positive and negative sentiment across all tweets. Each topic contains: (i) a set of entities, and (ii) the proportion by which an entity belongs to a topic. For example, entity *Greece* might belong by 70% to topic 1 and by 30% to topic 2.

**Fig 11:**
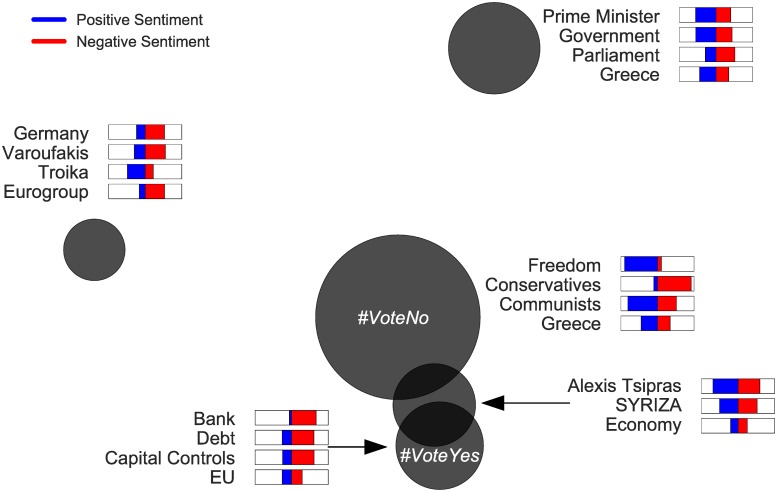
LDA topic model of referendum entities. Each circle is a topic placed according to PCA. Circle size is proportional to the marginal distribution of each topic. The 4 most frequent entities, that each topic contains, and their average positive sentiment (blue bars) and negative sentiment (red bars) are also shown.

**Fig 12:**
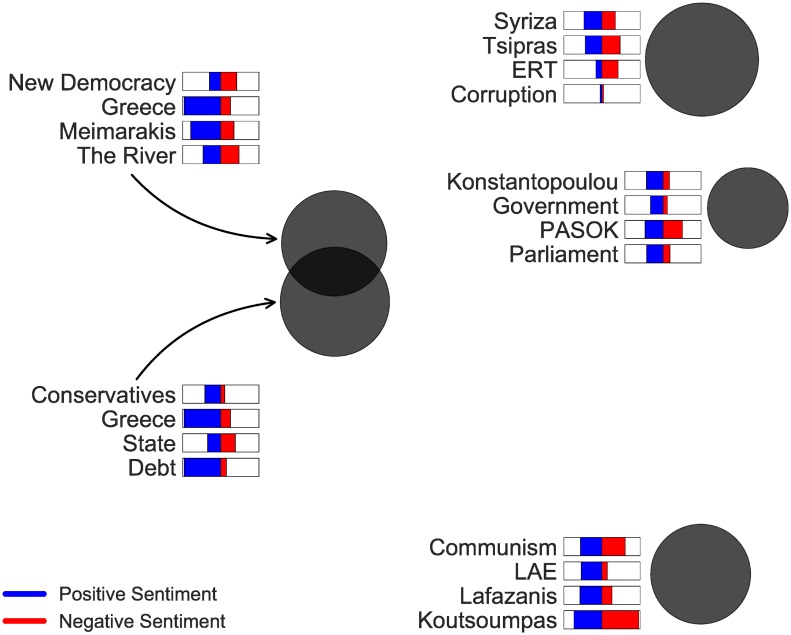
LDA topic model of elections entities. Each circle is a topic placed according to PCA. Circle size is proportional to the marginal distribution of each topic. The 4 most frequent entities, that each topic contains, and their average positive sentiment (blue bars) and negative sentiment (red bars) are also shown.

Figs 11 and 12 show the LDA topic analysis for referendum and elections, respectively.

In Fig 11 we notice that the topics containing *VoteYes* and *VoteNo* are unexpectedly close. Entities associated with the *VoteYes* topic contain stronger negative sentiments, whereas entities associated with the *VoteNo* topic contain stronger positive sentiments (except the *Conservatives* entity). Interestingly, the topic that contains the Prime Minister (Tsipras) and his leading party (SYRIZA) lie in the middle of the ‘YES’ and ‘NO’ topics despite the fact he and his party were strong ‘NO’ supporters. We also notice a topic on the left with prevalent negative sentiments that contain entities pervasive to the anti-austerity discourse like ‘Varoufakis’, ‘Troika’ and ‘Eurogroup’.

Regarding elections, in Fig 12 we observe that topics are placed according to the political spectrum. The two conjoined circles at the left represent conservative parties, whereas the rest three circles represent (i) the dominant center-left *SYRIZA* party at the top, (ii) politically center entities (namely, *PASOK*) in the middle, and (iii) the far-left parties at the bottom. One interpretation of this placing is that the conservative parties in Greece were more strongly affiliated than the left and center-left parties.

## Conclusion

We presented a detailed analysis of two Twitter datasets from two politically associated electoral events. Sentiment analysis and sarcasm detection were performed on the data in order to achieve high accuracy. Entity detection combined manual, semi-automatic and scripted processing as well as lexical resources to correctly assign sentiment. This combination was necessary for tackling the traditionally hard-to-analyze political domain by blending entity-level sentiment and data statistics.

Our results shed light on the often unnoticed societal and political trends that guide citizen choices and actions, which traditional polls fail to detect. The presented exploratory analysis revealed part of the public sentiment towards main entities along with their semantic proximities. This analysis was applied to two related electoral events, enabling the creation of lexical resources that covered the semantic content of a wide and complex political background. These resources, as an extension of the previously existing resources, were utilized for entity and sentiment detection, and are available both as general-purpose resources and, most importantly, optimized for the political domain. Although there is a plethora of studies in social networks regarding electoral or political events, most of these are concentrating on a specific aspect of the data (e.g., sentiment analysis, entity identification). Here, we argue that in-depth discovery in similar datasets should include at least 5 types of analysis: volume analysis, entity identification, sentiment detection, sarcasm correction and topic analysis. Results for each type of analysis can improve other types (for example, sentiment analysis enhancements can improve topic analysis). The analysis from this work aims to reveal quantitative aspects of data analytics that may prove helpful for political analysts and citizens alike. The semantic aspects of the results may be interpreted accordingly by the interested parties, making social interpretations on the qualitative level. Thus, while we are far from creating qualitative conclusions regarding the underlying social dynamics that affect a political discourse, we can certainly be assisted towards that goal by making sense of the vast amounts of social data through this approach.

The results also hinted further work. Since sentiment is a descriptive work for all emotions, and not all emotions are the same [56], an interesting next step for better understanding citizens and society, could be to detect emotion (sadness, happiness, fear, anger, etc.) and see how emotion drives societal and, consequently, political changes. Other interesting aspects that we plan to investigate in our future work are: (i) sentiment consistency (is there a specific sentimental spectrum for each user?); (ii) context-specific opinions (does sentiment give insights regarding opinions on certain entities?); and (iii) sentiments at the phrase or expression level (instead of per-word sentiment assignment, can we assign sentiment to the whole sentence by incorporating contextual subjectivity information?).

Finally, the presented analysis does not include methods to detect bots that are massively employed to spread content in favor of a specific candidate. This phenomenon has been detected in many elections (e.g., U.S presidential elections 2016 [57] and 2013 Australian Federal Election [47]) with yet unknown impact on the election results. Besides the significantly higher number of average tweets posted by “YES” voters in the referendum dataset, we do not have any other supporting indications that this phenomenon took place on the events that we analyzed.

To further improve our methods towards extracting qualitative insights regarding users’ political affiliations and in order to reveal potential malicious efforts to obscure the online discourse, we plan to analyze future electoral events mainly in the European area.
